# Supplementary material for: Identification of photoperiod-induced specific miRNAs in the adrenal glands of Sunite sheep (Ovis aries)
Source: Front Vet Sci. 2022 Jul 22;9:888207. doi: 10.3389/fvets.2022.888207 (PMC9354845; doi:10.3389/fvets.2022.888207)
Supplement: Supplementary file 1 [file Data_Sheet_1.ZIP › Supplementary file/miRDeep2 file.zip!/miRDeep2 file/Novel_Structure/SPLP42A3/Novel_338.pdf]

Provisional ID	:	NC_019464.2_15741
Score total	:	2.1
Score for star read(s)	:	-1.3
Score for read counts	:	0
Score for mfe	:	1.8
Score for randfold	:	1.6
Score for cons. seed	:	
Total read count	:	777
Mature read count	:	777
Loop read count	:	0
Star read count	:	0

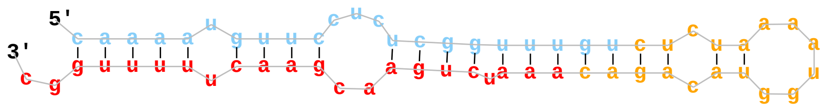

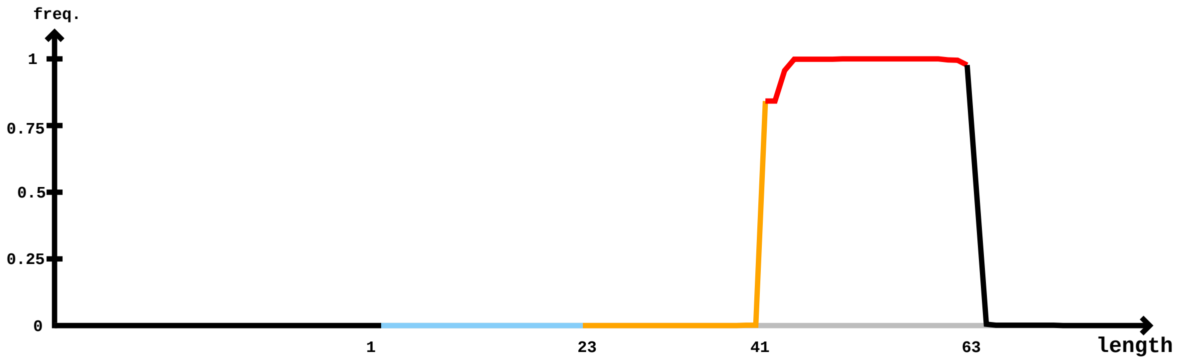


```
                                Star                                    Mature
5'- aaaacugaccuuuuccauuguauuggguuggccaaaauguuccucucgguuuguCucuaaaaugguacagacaaaucugaacgaacuuuuuggccaacccaaacuuccuugg   -3'    exp
    ..............(((..((.(((((((((((((((.((((...(((((((((((.((......)).))))))).))))..)))).)))))))))))))))))).....)))    reads   mm   sample
    ......................................................................acaaaucugaacgaacuuuuug....................    1       0    seq
    ........................................................................aaaucugaacAaacuuuuu.....................    3       1    seq
    ........................................................................aaaucugaacAaacuuuuugg...................    14      1    seq
    ........................................................................aaaucugaacAaacuuuuuggc..................    637     1    seq
    ..........................................................................aucugaacAaacuuuuuggc..................    16      1    seq
    ..........................................................................aucugaacAaacuuuuuggcc.................    73      1    seq
    ...........................................................................ucugaacAaacuuuuuggcc.................    30      1    seq
    ...........................................................................ucugaacAaacuuuuuggcca................    3       1    seq
    ................................................................................aUgaacuuuuuggccaacccaaa.........    1       1    seq
```
